# Unraveling Spatial Heterogeneity in Mass Spectrometry Imaging Data with GraphMSI

**DOI:** 10.1002/advs.202410840

**Published:** 2025-01-07

**Authors:** Lei Guo, Peisi Xie, Xionghui Shen, Thomas Ka Yam Lam, Lingli Deng, Chengyi Xie, Xiangnan Xu, Chris Kong Chu Wong, Jingjing Xu, Jiacheng Fang, Xiaoxiao Wang, Zhuang Xiong, Shangyi Luo, Jianing Wang, Jiyang Dong, Zongwei Cai

**Affiliations:** ^1^ Interdisciplinary Institute for Medical Engineering Fuzhou University Fuzhou 350108 China; ^2^ State Key Laboratory of Environmental and Biological Analysis Hong Kong Baptist University Hong Kong SAR 999077 China; ^3^ Department of Electronic Science Xiamen University Xiamen 361005 China; ^4^ School of Information Engineering East China University of Technology Nanchang 330013 China; ^5^ School of Business and Economics Humboldt‐Universitat zu Berlin 10099 Berlin Germany; ^6^ Department of Biology Hong Kong Baptist University Hong Kong SAR 999077 China; ^7^ College of Science Eastern Institute of Technology, Ningbo Ningbo 315000 China

**Keywords:** deep learning, graph convolutional network, mass spectrometry imaging, spatial heterogeneity

## Abstract

Mass spectrometry imaging (MSI) provides valuable insights into metabolic heterogeneity by capturing in situ molecular profiles within organisms. One challenge of MSI heterogeneity analysis is performing an objective segmentation to differentiate the biological tissue into distinct regions with unique characteristics. However, current methods struggle due to the insufficient incorporation of biological context and high computational demand. To address these challenges, a novel deep learning‐based approach is proposed, GraphMSI, which integrates metabolic profiles with spatial information to enhance MSI data analysis. Our comparative results demonstrate GraphMSI outperforms commonly used segmentation methods in both visual inspection and quantitative evaluation. Moreover, GraphMSI can incorporate partial or coarse biological contexts to improve segmentation results and enable more effective three‐dimensional MSI segmentation with reduced computational requirements. These are facilitated by two optional enhanced modes: scribble‐interactive and knowledge‐transfer. Numerous results demonstrate the robustness of these two modes, ensuring that GraphMSI consistently retains its capability to identify biologically relevant sub‐regions in complex practical applications. It is anticipated that GraphMSI will become a powerful tool for spatial heterogeneity analysis in MSI data.

## Graphical abstract

Mass spectrometry imaging (MSI) provides valuable insights into metabolic heterogeneity within biosamples. GraphMSI is proposed, a deep learning‐based method that integrates metabolic profiles with spatial information to improve the analysis of spatial heterogeneity. GraphMSI leverages biological context and enhances three‐dimensional segmentation through two optional enhanced modes: scribble‐interactive and knowledge‐transfer. This innovative approach positions GraphMSI as a robust and versatile tool for MSI data analysis.

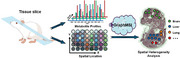

## 1 Introduction

Understanding spatial heterogeneity within biological tissues is essential for deciphering the mechanisms of various biological processes.^[^ ^1^, ^2^ ^]^ Spatial omics techniques, which employ in situ molecular measurements, have been instrumental in revealing the intricate spatial heterogeneity within tissues.^[^ ^3^, ^4^ ^]^ Among these techniques, mass spectrometry imaging (MSI) stands out as a label‐free method that enables comprehensive spatial profiling of thousands of molecules, offering critical insights into metabolic heterogeneity within tissue.^[^ ^5^, ^6^, ^7^, ^8^ ^]^ Due to its high sensitivity and high throughout, MSI has been widely used to explore the relationship between spatial heterogeneity and disease progression, highlighting its importance in contemporary biomedical research.^[^ ^9^, ^10^ ^]^

Spatial segmentation is a crucial step in analyzing heterogeneity in MSI data.^[^ ^11^ ^]^ It involves partitioning the mass spectra into distinct clusters associated with different pathological or physiological categories. Effective spatial segmentation can reveal the landscape of heterogeneous tissue samples, facilitating a deeper understanding of the biological processes. However, spatial segmentation remains challenging due to the complexities of MSI data, which are characterized by high dimensionality, low signal‐to‐noise ratio, and a lack of benchmark datasets.^[^ ^12^ ^]^ Segmentation methods are typically classified as either supervised or unsupervised, depending on whether the ground truth data is used. Supervised methods often rely on reference images from other modalities, such as hematoxylin and eosin (H&E)‐stained image or magnetic resonance imaging, to guide MSI data segmentation.^[^ ^13^, ^14^ ^]^ However, because MSI data often contains much richer molecular information than these other imaging modalities, using them as guides can obscure “hidden” structures within tissue, leading to biased spatial heterogeneity analysis.^[^ ^15^, ^16^ ^]^ Unsupervised methods, on the other hand, cluster spots based on their spectral similarity and spatial proximity, making them more practical for spatial heterogeneity analysis when reliable ground truth is often unavailable.

Several unsupervised methods have been developed specifically for spatial segmentation of MSI data. For example, Abdelmola et al. combine t‐distributed stochastic neighbor embedding (t‐SNE) with K‐Means method to analyze tumor heterogeneity, identifying several molecular markers associated with prognostic tumor subpopulations.^[^ ^17^ ^]^ Additionally, vender software SCiLS Lab and widely used Cardinal package enhance spatial measurement by incorporating spatial location, thereby reducing the occurrence of undesirable discontinuous results.^[^ ^18^, ^19^ ^]^ Despite these advancements, most of these methods rely on statistical model‐based algorithms that depend on specific mathematical assumptions about MSI data. Given the high heterogeneity of MSI data, different regions may have varying degrees of validities under a given model‐based clustering algorithm,^[^ ^20^ ^]^ often resulting in poor‐determined segmentation results. Deep learning, with its data‐driven strategy and the ability of automatically capture heterogeneous structure in MSI data, offers a more flexible and adaptive strategy for MSI analysis.^[^ ^21^, ^22^, ^23^ ^]^ For example, Gardner et al. develops a convolutional autoencoder (CNNAE) model specifically for MSI data, which outperforms statistical model‐based methods in spatial heterogeneity analysis.^[^ ^24^ ^]^ Similarly, Kim et al. presents a standard convolutional neural network (CNN)‐based unsupervised segmentation method that delivers more accurately results than model‐based methods, and it can be directly applied to MSI segmentation.^[^ ^25^ ^]^

Nevertheless, existing methods still face significant challenges in practical biomedical research, highlighted by the following issues: First, improper use of spatial information can suppress small but critical biological signals, and may even introduce artificial non‐biological signals that negatively affect segmentation results.^[^ ^26^ ^]^ Second, commonly used unsupervised segmentation methods often produce algorithmically correct but biologically inappropriate segmentation. For example, sub‐regions with histomorphological differences are often incorrectly merged into a single region when MSI data fails to capture these differences or when the differences are too subtle for unsupervised methods to detect.^[^ ^27^ ^]^ Third, the three‐dimensional (3D) MSI data provides rich molecular information on high chemical specificity across multiple tissue sections. However, commonly used methods for analyzing 3D MSI data are hindered by batch effects among different slices and significant computational demand on hardware.^[^ ^28^ ^]^ There is an urgent need for more efficient methods that allow researchers to analyze large 3D MSI data with enhanced speed and accuracy.

To address these challenges, we propose a novel deep learning‐based method, namely GraphMSI, specifically designed for unraveling spatial heterogeneity within tissue. GraphMSI comprises two key modules: dimensionality reduction (DR) and feature clustering (FC). The DR module employs parametric‐UMAP to reduce data dimensionality while preserving spectral information and minimizing noise. In the FC module, the graph convolutional network (GCN) is used instead of the commonly used CNN to learn more meaningful representation for each spot based on its metabolic profiles and spatial location. To reduce the computational burden of GCN, we limit the construction of the adjacency matrix to consider the feature difference between each spot and its 8 nearest neighbors (rather than all spots), as illustrated in Figure S1 (Supporting Information). Comparative experiments and ablation study on mouse kidney dataset demonstrate the effectiveness of GraphMSI, outperforming commonly used methods in both visual inspection and quantitative evaluation while maintaining acceptable computational complexity.

Existing methods face challenges due to inadequate integration of biological context and excessive computational requirements. To address these issues, GraphMSI can be extended to two operational modes, scribble‐interactive and knowledge‐transfer to ensure versatile applications across various biomedical scenarios. The scribble‐interactive mode allows for the incorporate of partial or coarse biological contexts, enabling corrections of inappropriate segmentations produced by the basic GraphMSI model. Meanwhile, the knowledge‐transfer mode facilitates the segmentation of unseen MSI data by utilizing the model pre‐trained on MSI data from adjacent slice, allowing for faster and more accurate 3D MSI segmentation. Two typical applications illustrate the effectiveness of GraphMSI in these two modes: pinpointing organs or sub‐organs in mouse fetus dataset to improve segmentation results, and more effective segmentation of heterogeneous regions in complex 3D cancer cell spheroids (CCS) dataset. GraphMSI is expected to become a critical tool for exploring spatial heterogeneity in MSI data.

## 2 Results

### 2.1 Overview of GraphMSI Model

**Figure 1:**
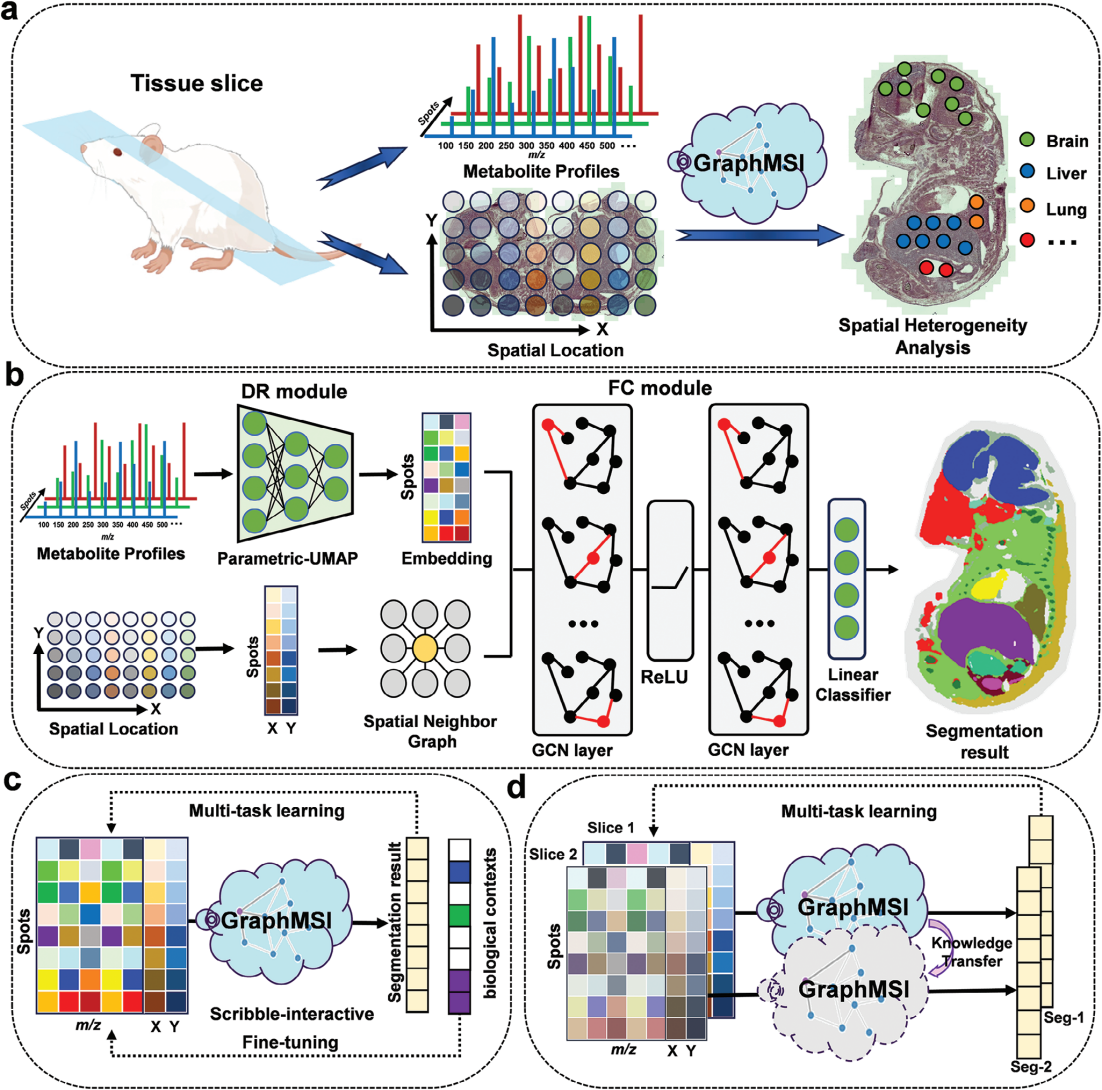
Overflow of the proposed GraphMSI for MSI segmentation. a) GraphMSI is designed to integrate the metabolite profiles and spatial location for each spot to generate the segmentation result for spatial heterogeneity analysis. b) GraphMSI takes as inputs the MSI data that includes the metabolite profiles and spatial location. Latent embedding data is obtained using parametric‐UMAP to preserve the informative features from the metabolite profiles. Then, the spatial neighborhood graph is constructed based on the spot coordinates. Both of them are inputted into the two GCN layers and a classifier to obtain the spatial segmentation result. c) The GraphMSI model is trained using the multi‐task learning. Particularly, the scribble‐interactive mode incorporates the partial or coarse biological contexts to fine‐tuning the basic model to achieve enhanced results. d) The GraphMSI with knowledge‐transfer mode trains on MSI data from slice‐1 and then directly applies the trained model to perform segmentation on unseen MSI data from slice‐2, which are adjacent slices, without the need for re‐training.

The workflow of GraphMSI is illustrated in **Figure** **1**. Biological tissue is first sectioned, followed by the application of the MSI technique to measure the metabolic profiles and spatial location for each spot. These data are then input into GraphMSI for spatial heterogeneity analysis within the tissue, as shown in Figure 1a. The GraphMSI model consists of two modules: the DR module and the FC module. The DR module employs parametric‐UMAP which incorporates multiple fully connection networks to preserve spectral information while minimizing noise. The FC module, built on a GCN‐based architecture,^[^ ^29^ ^]^ includes two GCN layers and a classifier to achieve accuracy segmentation by effectively capturing spectral and spatial information from MSI data, as depicted in Figure 1b. A multi‐task learning loss function is specially designed to ensure that GraphMSI produces reliable segmentation results, with the backpropagation of errors illustrated in Figure S2 (Supporting Information). Detailed descriptions of the model architecture and training scheme can be found in the Experimental Section.

To enhance the practical applicability of GraphMSI, it can be extended to two optional modes: scribble‐interactive and knowledge‐transfer, thereby increasing its adaptability to various scenarios. The scribble‐interactive mode, shown in Figure 1c, improves basic segmentation by incorporating scribbles input that integrate partial or coarse biological contexts from other sources, such as reference imaging modalities or domain knowledge. Meanwhile, the knowledge‐transfer mode, illustrated in Figure 1d, enables segmentation on unseen MSI data by using a model pre‐trained on adjacent slice's data, eliminating the need for re‐training. This significantly improves the speed and accuracy of 3D MSI segmentation. Notably, these two modes are complementary and can be used together in complex application scenarios.

### 2.2 Precisely Resolving Mouse Kidney Structures Using GraphMSI

**Figure 2:**
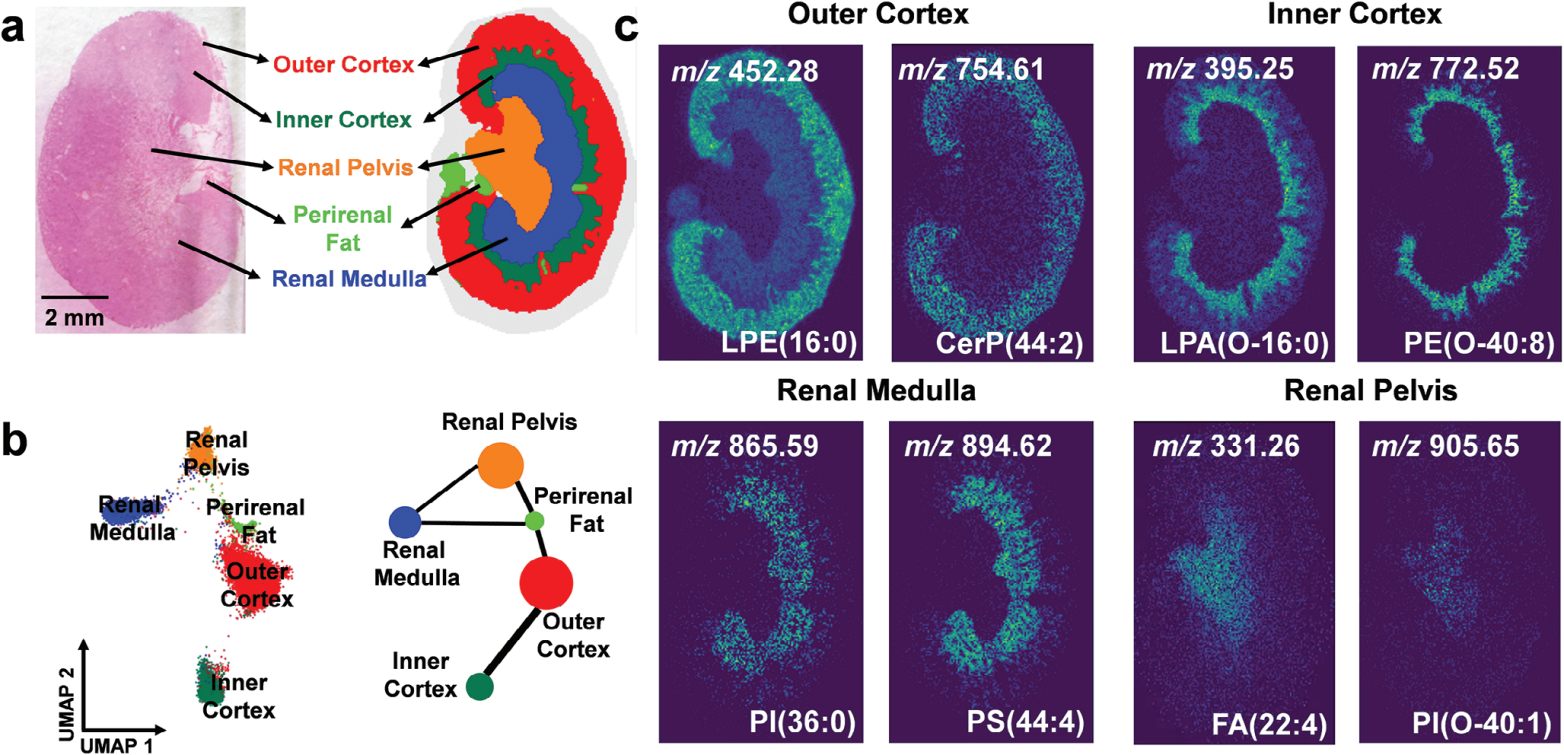
Results of GraphMSI on mouse kidney dataset. a) Color‐encoded segmentation result derived from GraphMSI alongside the corresponding H&E‐stained image. b) Scatter plot of data points in the UMAP embedding space related to (a) displayed on the left panel, and a network depicting metabotypic similarities across sub‐regions on the right panel. c) Identification of potential molecular markers associated with four kidney sub‐regions.

Differentiating kidney sub‐regions based on metabolic heterogeneity is critical for understanding the links between specific functions and their corresponding metabolic profiles.^[^ ^30^, ^31^ ^]^ In this study, GraphMSI is applied to an MSI dataset of the mouse kidney (**Figure** **2**). The segmentation results reveal five distinct sub‐regions: the outer cortex (red), inner cortex (dark green), renal medulla (blue), renal pelvis (orange), and an additional perirenal fat region (light green), as shown in Figure 2a. These sub‐regions are consistently observed in the corresponding H&E‐stained images.

Next, the metabotype similarity among different kidney sub‐regions kidney is analyzed (Figure 2b). The scatter plot shows distinct separation of data points in the embedding space, indicating the significant differences in metabolic profiles across these sub‐regions. A metabotypic similarity network is then constructed by calculating the Euclidean distance between cluster centers from different regions. The results demonstrate that spatially adjacent kidney sub‐regions exhibited similar metabolic features, with the renal pelvis showing high similarity to the medulla, and the outer cortex resembling the inner cortex. These findings align with previous studies,^[^ ^32^, ^33^ ^]^ confirming the capability of GraphMSI in exploring the biological function of mouse kidney sub‐regions.

Potential markers for each sub‐region are screened, with some manually annotated by matching known metabolites from public databases within the 5 ppm tolerance (Figure 2c). Notably, high expression levels of *m/z* 452.28 LPE(16:0) and *m/z* 754.61 CerP(44:2) are found in the outer cortex; *m/z* 395.25 LPA(O‐16:0) and *m/z* 772.52 PE(O‐40:8) are detected in the inner cortex; *m/z* 865.59 PI(36:0) and *m/z* 894.62 PS(44:4) in the renal medulla; *m/z* 905.65 PI(O‐40:1) and *m/z* 709.51 PA(O‐38:4) in the renal pelvis. These markers have been reported in previous study.^[^ ^34^ ^]^ Additional co‐localized ions identified through searches are presented in Figure S3 (Supporting Information). These results demonstrate the potential of the proposed GraphMSI approach for unrevealing metabolic heterogeneities in the mouse kidney MSI dataset.

### 2.3 Using GraphMSI with Scribble‐Interactive Mode for Comprehensive Capture of Mouse Fetus Anatomy

Accurately distinguishing organ and sub‐regions is complex but essential preprocessing step with important applications in embryological genetics, pathology, and pharmacology.^[^ ^35^, ^36^ ^]^ Due to the heterogeneity in mouse fetus tissue, current segmentation methods often fail to delineate organs and sub‐regions effectively. The introduction of GraphMSI with its scribble‐interactive mode bridges this gap, enabling comprehensive mapping of the mouse fetus anatomy by incorporating the coarse and partial biological contexts from H&E‐stained image.

**Figure 3:**
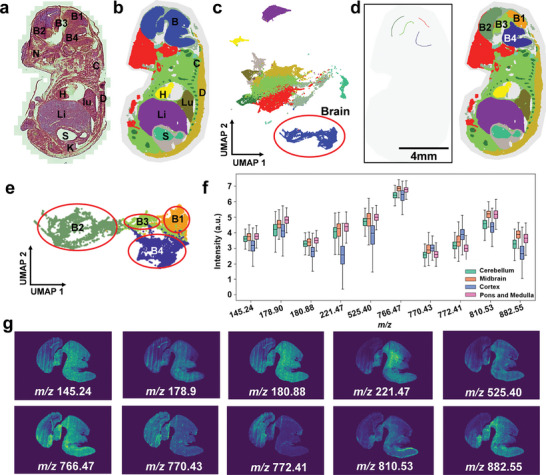
Enhanced segmentation results on brain region in mouse fetus dataset using GraphMSI with scribble‐interactive mode. a) H&E‐stained image; b) Color‐encoded segmentation result derived from the basic of GraphMSI using unsupervised learning manner; c) Scatter plot of the whole data points in the UMAP embedding space related to (b); d) The created scribble and the corresponding segmentation results using scribble‐interactive mode; e) Scatter plot of the brain data points related to (d); f) Boxplot of Top 10 discriminative ions whose AUC > 0.70; g) Spatial distribution of discriminative ions.

**Figure** **3** showcases the capabilities of GraphMSI with the scribble‐interactive mode for segmenting MSI dataset of the mouse fetus. Twelve organs/sub‐organs can be clearly observed from H&E‐stained image,^[^ ^37^ ^]^ including cerebellum (B1), midbrain (B2), cortex (B3), and pons and medulla (B4), nasal cavity (N), cartilage (C), lung (Lu), stomach (S), dorsal skin (D), heart (H), Liver (Li) and kidney (K) (the left panel of Figure 3a). Using these organ identification as biological contexts, seven organs/sub‐organs — brain (B), cartilage (C), lung (Lu), stomach (S), dorsal skin (D), heart (H), and Liver (Li)— are successfully segmented by the basic GraphMSI model (Figure 3b). However, some algorithmically correct but biologically inappropriate segmented regions are identified (Figure 3c). For example, while clustering spots with similar metabolic profiles in the brain region is algorithmically accurate from a global perspective, the sub‐regions of the cerebellum (B1), midbrain (B2), cortex (B3), and pons and medulla (B4) could not be distinguished, which is biological inappropriate. It suggests that the basic of GraphMSI, which employs an unsupervised learning approach, lacks sufficient local adaptability, thereby limiting the analysis of metabolic heterogeneity between different brain sub‐regions. GraphMSI with scribble‐interactive mode can correct these inappropriate segmentations. By using sub‐organ information from H&E‐stained image as biological contexts, such as the mouse brain consisting of at least four sub‐organs — cerebellum (B1), midbrain (B2), cortex (B3), and pons and medulla (B4) — we can refine the segmentation. In this case, a blank scribble image is created, and four distinct color scribbles are drawn to correspond to these sub‐regions (Figure 3d). Using the scribble‐interactive mode, GraphMSI successfully segments the mouse brain into four distinct sub‐regions (B1, B2, B3, and B4), accurately matching the cerebellum, midbrain, cortex, and pons and medulla observed in histological images. Figure 3e shows these four regions clearly separated in UMAP embedding space using scribble‐interactive mode.

To further illustrate the significant differences between the identified sub‐regions, the area under the curve (AUC) is used to identify the discriminative ions for each region, with top 10 ions (AUC ≥ 0.70) displayed in Figure 3f. Among these, *m/z* 882.55 (AUC = 0.78), *m/z* 810.53 (AUC = 0.78), *m/z* 221.47 (AUC = 0.75), *m/z* 772.41 (AUC = 0.73), *m/z* 525.40 (AUC = 0.72), *m/z* 180.88 (AUC = 0.72), *m/z* 766.47 (AUC = 0.71), *m/z* 145.24 (AUC = 0.71), *m/z* 178.90 (AUC = 0.70) and *m/z* 770.43 (AUC = 0.70) are identified as differentially expressed across these regions (Figure 3g), some of them are manually assigned by matching the known metabolites from public databases, as shown in the Table S1 (Supporting Information). Additionally, improvements in kidney and nasal cavity segmentation achieved by GraphMSI with the scribble‐interactive mode are illustrated in Figures S4 and S5 (Supporting Information). These findings highlight the generalizability of GraphMSI with scribble‐interactive mode in refining algorithmically correct but biologically inappropriate results.

**Figure 4:**
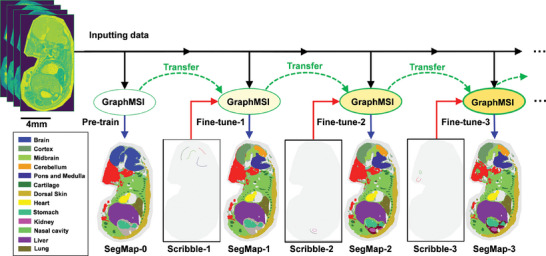
The schematic of the iterative fine‐tuning process in scribble‐interactive mode. The process begins with the input of preprocessed MSI data into GraphMSI in an unsupervised manner, generating an initial segmentation (SegMap‐0) that identifies major anatomical regions, such as the brain, cartilage, dorsal skin, heart, stomach, liver, and lung. Next, Scribble‐1 provides detailed guidance to refine SegMap‐0 by focusing on specific brain regions: the cortex, midbrain, cerebellum, and pons and medulla, resulting in SegMap‐1. Additional fine‐tuning steps incorporate Scribble‐2, which targets the kidney and other abdominal regions, yielding SegMap‐2, and Scribble‐3, which identifies the nasal cavity and neck regions, culminating in SegMap‐3.

The flexibility of GraphMSI with scribble‐interactive mode is further demonstrated by its ability to enhance segmentation results through multiple interactive refinements, as seen in our previous work.^[^ ^38^ ^]^ As shown in **Figure** **4**, three scribble images (Scribble‐1, Scribble‐2, and Scribble‐3) are used sequentially to fine‐tune the GraphMSI model. Each scribble image is designed to correct specific regions: Scribble‐1 divides the brain into four sub‐regions, Scribble‐2 differentiates the kidney from the abdominal area, and Scribble‐3 separates the nasal cavity from the neck region. Initially, the base GraphMSI model uses the inputting data to create a preliminary segmentation result (SegMap‐0). Fine‐tuning with Scribble‐1, Scribble‐2, and Scribble‐3 subsequently produces the enhanced segmentation results SegMap‐1, SegMap‐2, and SegMap‐3, respectively. The transfer of the model between iterations ensures versatility in the results. These findings highlight the adaptability of GraphMSI with scribble‐interactive mode, emphasizing its practical application in biological research.

In addition, the robustness of the scribble‐interactive mode is discussed in Section S1 (Supporting Information). The results indicate that GraphMSI consistently retains its capability to identify biologically relevant sub‐regions in complex practical applications.

### 2.4 Accelerated 3D Segmentation of Cancer Cell Spheroids Using GraphMSI with Knowledge‐Transfer Mode

The application of MSI segmentation on 3D CCS dataset is critical for advancing our understanding of the tumor microenvironment and its spatial heterogeneity.^[^ ^39^, ^40^ ^]^ Traditional segmentation methods for 3D MSI dataset are often limited by high computational demands and the batch effect across the tissue slices. Here, we utilize the knowledge‐transfer mode of GraphMSI to segment 3D CCS dataset comprising 27 slices, demonstrating its capability in handling 3D MSI data.

**Figure 5:**
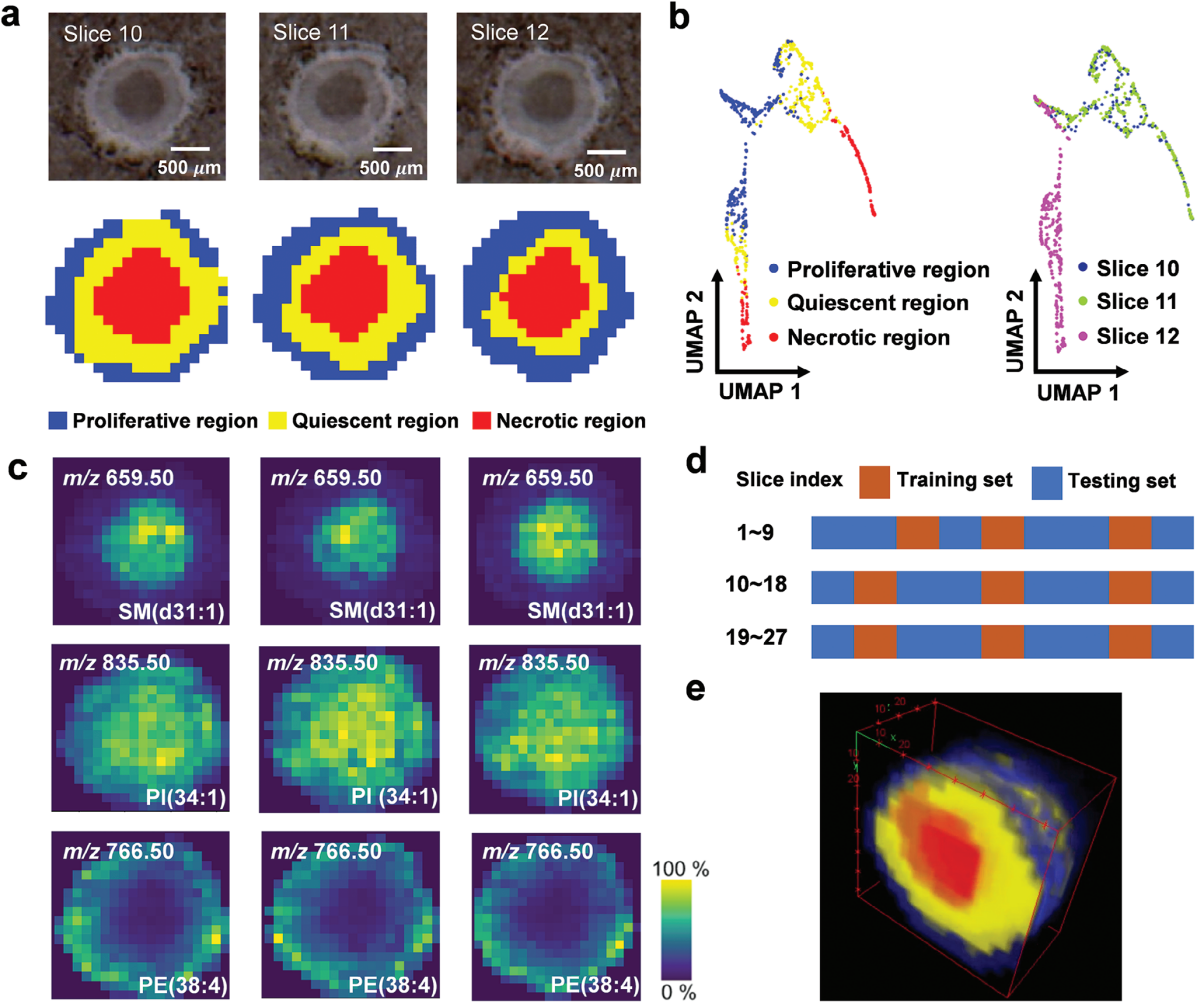
Results of the GraphMSI with knowledge‐transfer mode on 3D CCS dataset. a) Segmentation of slices 10, 11, and 12 along with the corresponding optical image; b) Scatter plot of data points in the UMAP embedding space, colored according to (a) on the left panel, and colored by slice index on the right panel; c) Identification of potential molecular markers associated with each sub‐region. d) Division of the training and testing sets across all slices for GraphMSI model; e) The result of 3D reconstruction for all 27 slices.

Figure S6 (Supporting Information) shows the optical images of 27 slices, while Figure S7 (Supporting Information) presents a scatter plot in UMAP space, highlighting batch effects across the slices and the complexities in analyzing 3D CCS MSI dataset. In **Figure** **5**a, we demonstrate a segmentation where MSI data from slice 11 is used for model training, and the pre‐trained model is then applied to predict segmentation in adjacent slices 10 and 12, facilitating knowledge‐transfer between slices. The segmentation clearly delineates three distinct regions within the 3D CCS: a proliferative region (blue), a quiescent region (yellow), and a necrotic region (red), all consistent with morphological evaluations. The scatter plot in the left panel of Figure 5b shows that metabolic features from these regions are distinct, with clear separations in the embedding space. Notably, we find that data points from slice 12 are distinctly separated from those of slices 10 and 11 (right panel of Figure 5b). An analysis of variance (ANOVA) test reveals that slice 12 exhibits significant metabolic differences compared to slices 10 and 11 (*p*‐value < 0.05), indicating the batch effect among the slices, as shown in Table S2 (Supporting Information). The GraphMSI model alleviates the batch effect by focusing on modeling relationships between spots rather than relying solely on basic intensity information.

Figure 5c highlights the potential molecular markers identified in these regions: high expression of *m/z* 659.50 SM(d31:1) in the necrotic region; *m/z* 835.50 PI(34:1) in both the quiescent and necrotic regions; and *m/z* 766.50 PE(38:4) in the proliferative region, consistent with the previous studies.^[^ ^41^, ^42^ ^]^ Figure 5d shows the distribution of the training and testing set, with 33.33% of the dataset (spectra from 9 tissue sections) used for training and 66.67% (spectra from 18 tissue sections) for testing, saving approximately two‐thirds of the computational time compared to training on all slices individually, as shown in Table S3 (Supporting Information). Segmentation results, UMAP scatter plots, and spatial distribution of potential molecular makers for all slices are shown in Figures S8–S12 (Supporting Information), showing that the GraphMSI with knowledge‐transfer mode accelerates the analysis process while maintaining segmentation quality. The 3D reconstruction of these 27 slices using ImageJ is displayed in Figure 5e. Overall, these results illustrate the effectiveness of GraphMSI with knowledge‐transfer mode in performing MSI segmentation on unseen data, significantly speeding up the 3D segmentation process and reducing the impact of batch variations.

## 3 Discussion

### 3.1 GraphMSI Surpasses Commonly Used Methods in Both Visual Inspection and Quantitative Evaluation

In this study, GraphMSI is compared with commonly used methods for analyzing MSI dataset of mouse kidney (Figure S13a, Supporting Information), including t‐SNE + K‐Means,^[^ ^17^ ^]^ SCiLs Lab,^[^ ^18^ ^]^ Cardinal,^[^ ^19^ ^]^ CNNAE + K‐Means,^[^ ^24^ ^]^ and a CNN‐based segmentation method.^[^ ^25^ ^]^ To ensure fair comparison across all methods, the number of clusters is manually calibrated to delineate five key anatomical regions: outer cortex, inner cortex, renal medulla, renal pelvis, and perirenal fat. The segmentation result from t‐SNE+ K‐Means, which do not incorporate spatial information, is marred by numerous discrete points and noise, making sub‐regions difficult to delineate clearly. In contrast, the SCiLs Lab, Cardinal, CNNAE + K‐Means, and CNN‐based segmentation method produce more continuous segmentation results, successfully identifying the five regions. However, these methods also introduce edge artifacts due to the improper handling of spatial information, as illustrated in Figure S14 (Supporting Information). GraphMSI, on the other hand, generates continuous segmentation results without edge artifacts, providing superior visual clarity in the analysis of kidney dataset. Additionally, we quantify the effectiveness of each segmentation method by measuring the degree of co‐localization between the four segmented regions and their corresponding manually annotated lipids, LPE(16:0), LPA(O‐16:0), PS(44:4), and PI(40:1), as shown in Figure S13b (Supporting Information). The AUC, as described in the Experimental Section, is used for quantitative evaluation. The proposed GraphMSI model achieves the highest AUC value, demonstrating its superior effectiveness in MSI segmentation at a quantitative level.

### 3.2 The Superior Performance of GraphMSI is Driven by Its GCN‐Based Architecture and Multi‐Task Learning

To investigate the factors contributing to the superior performance of GraphMSI, we conduct the ablation studies to explore the model's internal workings. First, unlike previous deep learning‐based segmentation methods that use CNNs for feature extraction, GraphMSI employs GCNs to capture both spectral and spatial information from MSI data. Figure S15 (Supporting Information) compares the segmentation results obtained using GCN and CNN. Notably, the GCN approach significantly reduces the artificial edge effects seen in the CNN version, demonstrating GCN's adaptability in processing spatial information. Additionally, the multi‐task learning loss function, specifically designed for GraphMSI ensures the production of reliable segmentation results. Figure S16 (Supporting Information) presents the segmentation results when each individual loss component is removed from the multi‐task leaning loss function. When $L_{umap}$ is removed, only the renal pelvis is identified, with other regions significantly distorted, highlighting $L_{umap}$’s role in preserving the rich metabolic profiles during model training. Exclusion $L_{sim}$ results in numerous unknown regions, indicating the model's difficulty in clustering similar spots into the same region. The removal of $L_{tv}$ leads to discontinuous segmentation, demonstrating ${L_{tv}}^{\prime }s$ ability to reduce non‐biological discrepancies between spatially adjutant spots. The absence of $L_{ent}$ results in the segmentation with only one region, demonstrating its importance in preventing travail solution. Finally, removing $L_{scr}$ inherent hinders the model from learning any biological contexts from the scribble‐input. These findings demonstrate the GCN‐based architecture and multi‐task learning are critical to the superior performance of GraphMSI.

## 4 Conclusion

We present the GraphMSI, a deep learning‐based method designed for MSI data to elucidate spatial heterogeneity within tissue. Multiple results have demonstrated that GraphMSI is a straightforward and more accurate method for identifying sub‐regions with different metabolic profiles, offering more reliable segmentation results compared to commonly used methods. The ablation study confirms that the effectiveness of GraphMSI stems from its GCN‐based architecture combined with a multi‐task learning training strategy. The GCN‐based architecture not only preserves essential information but also prevents the creation of edge artifacts in contiguous segmentation results. Additionally, the implementation of multi‐task learning ensures that GraphMSI delivers reliable segmentation results.

GraphMSI can be further extended to two enhanced optional modes: scribble‐interactive and knowledge‐transfer, highlighting its superior flexibility and effectiveness compared to commonly used methods. The versatility and robust performance of these modes are demonstrated across two representative MSI datasets: the whole mouse fetus and 3D CCS. In the scribble‐interactive mode, the model leverages partial or coarse biological contexts from reference images or domain knowledge, enabling iterative refinements that enhance segmentation accuracy and biological relevance for complex dataset like the whole mouse fetus. The knowledge‐transfer mode allows the model to segment new, unseen MSI data by utilizing a network pre‐trained on adjacent slices, eliminating the need for re‐training and mitigating batch effects, particularly in the analysis of 3D CCS. These modes significantly enhance the versatility of GraphMSI, making it become a more practical tool for a variety of MSI applications.

However, a limitation of GraphMSI is that the user‐defined cut‐off value for graph construction can impact segmentation results, with inappropriate cut‐off values leading to suboptimal results. To address it, we develop a cut‐off selection strategy and an interactive graphical user interface for GraphMSI to assist in determining appropriate cut‐off values for effective segmentation, as detailed in Section S2 (Supporting Information). Furthermore, the computational time for the GCN‐based model increases more significantly than that of CNN‐based models as the data size grows due to the tensor operations involving the adjacency matrix (as shown in Table S4, Supporting Information), posing a challenge when applying GraphMSI to large‐scale MSI data analysis. Future studies may explore more advanced architectures to improve the computational efficiency of the GraphMSI model. Since GraphMSI does not require prior knowledge about specific molecular characteristics, it is anticipated to have broad applicability across various computational tasks involving other spatial omics techniques, including spatial transcriptomics data, spatial proteomics, and other forms of medical imaging. GraphMSI is expected to become a general tool for exploring spatial heterogeneity in spatial omics data.

## 5 Experimental Section

### Samples Collection and Data Acquisition

All animal experiments conducted in this study received approval from the Committee on the Use of Human and Animal Subjects in Teaching and Research at Hong Kong Baptist University (approval number: REC/22‐23/0468) and relied on by all animal experiments. Three typical MSI datasets including mouse kidney, mouse fetus, and 3D CCS are employed to comprehensively evaluate and validate the performance of the proposed GraphMSI method. Details of the sample preparation and data acquisition for these three datasets can be found in Sections S3 and S4 (Supporting Information). Then, the raw MSI data were exported from the MSI instrument.

### Data Preprocessing

Data preprocessing was performed to improve the MSI data by decreasing the unwanted effects introduced during sample preparation and data acquisition. Specifically, the baseline correlation, peak finding, and peak alignment were performed using SCiLs Lab (Bruker company, Germany). Peak filtering was performed using Python scripts. Finally, the raw MSI data was converted to preprocessed matrix **M**_X*Y*Z_, where the *X* and *Y* represent the horizontal and vertical number of spectra, and the *Z* represents the number of detected ions.

### Architecture of GraphMSI

By introducing the deep learning into the MSI segmentation, the GraphMSI can cluster the spot with similar metabolite profile and spatial location into the same cluster. It consists of dimensionality reduction (DR) and feature clustering (FC) module. DR module is to learn the mapping function *f*(· |**θ**) to project the high‐dimensional MSI data **M**_X*Y*Z_ to the low‐dimensional embedding data **E**_X*Y*l_, as follows:

(1) $$E_{X*Y*l}=f_{\theta }\;\left(M_{X*Y*Z}\right)$$

where **θ** is the network parameter to be trained. DR module consists of two fully connection layers, where the BatchNorm layer and ReLU activation function are applied in the first layer, while no activation function was used in the second layer. FC module was achieved by learning the nonlinear mapping function *g*(· |ϑ) to cluster the embedding data **E**_X*Y*l_ into the segmentation result **O**_X*Y_, as follows:

(2) $$O_{X*Y}=g_{\vartheta }\;\left(E_{X*Y*l}\right)$$

where ϑ is the network parameter to be trained. There are many structures that can be selected for MSI unsupervised segmentation. In this study, GCNs followed by the *argmax* classifier were adopted here for obtaining robust, stable, and accuracy segmentation results. Specifically, the graph G=(V,E) was first constructed using the spectral and spatial information of the MSI data. In this graph G=(V,E), V represents the set of nodes, where each node corresponds to the embedding data of an individual spot. The set E consists of edges that represent the connections within the graph, linking the nodes based on their spectral and spatial similarity. For node $v_{u}\in \;V$, to relieve the computational complexity of graph construction, the focus was only on the differences between *v_u_* and its 8‐neighbor spots U(*v_u_*). The edge between *v_u_* and its neighbor node *v_i_* ∈  U(*v_u_*) is established if the Euclidean distance within the user‐defined cut‐off value, and the adjacency matrix **A** of graph G is defined as follows:

(3) $$a_{ui}=\left\{\begin{array}{c} 1\;if\;dist\left(v_{u},v_{i}\right)<\mathrm{cut}-\mathrm{off}\;\mathrm{value}\\ 0\;if\;dist\left(v_{u},v_{i}\right)\geq \mathrm{cut}-\mathrm{off}\;\mathrm{value}\; \end{array}\right.$$

Then, the graph G=(V,E) is inputted into two GCN layers to aggregate the neighborhood information for each node, thus offering the flexibility of feature‐specific aggregation of information provided by neighboring spectra. Here, the operation in two GCN layers are displayed as follows:

(4) $$H^{\left(1\right)}=\mathrm{ReLU}\left(\hat{A}\hat{E}W^{\left(0\right)}\right)$$

(5) $$H^{\left(2\right)}=\hat{A}H^{\left(1\right)}W^{\left(1\right)}$$

where the $\hat{A}={\hat{D}}^{-\frac{1}{2}}\;\left(A+I\right){\hat{D}}^{-\frac{1}{2}}$, $\hat{E}\in R^{XY\times l}$ is the 2D matrix obtained by reshaping **E** ∈ *R* ^*X* × *Y* × *l*^, $\hat{D}$ is the diagonal degree matrix of (**A** + **I**), **I** is the identity matrix, **W** ^(0)^ ∈ *R* ^*l* × *k*^ and **W** ^(1)^ ∈ *R* ^*k* × *k*^ are the parameters that need to be trained. Finally, the output **H** ^(2)^ is reshaped to the response map **R**_X*Y*k_ = ( *r*_*x*, *y*, *i*_) , and it is inputted to BatchNorm layer followed by the *argmax* classifier to get the segmentation result **O**_X*Y_ = (*O*_*x*, *y*_) , as follows:

(6) $$o_{x,y}:=\left\{i\left|\;r_{x,y,i}\geq \;r_{x,y,j},\forall j\neq i\leq k\right.\right\}$$

### Training Scheme and Implementation

The DR module was implemented leverages an advanced unsupervised dimensionality reduction technique, uniform manifold approximation and projection (UMAP), which has consistently outperformed other methods such as PCA and t‐SNE in handling MSI data.^[^ ^43^ ^]^ Here, the enhanced variant, termed parameter‐UMAP was presented to overcome the two major limitations commonly associated with the standard UMAP technique: 1) the challenge of accurately projecting unseen data into a low‐dimensional space without resorting to approximations, 2) the risk of producing variable embedding result for the same input data due to the non‐convex nature of the UMAP loss function. Specifically, the core objective of the parameter‐UMAP was to establish a non‐linear mapping from the original high‐dimensional feature space to a low‐dimensional (often 20‐dimensional) latent space. This mapping was determined based on the mutual similarities among data points in the high‐dimensional context. The loss function of parameter‐UMAP as follows:

(7) $$\;L_{DR}=\sum_{i\neq j}p_{i,j}log\frac{p_{i,j}}{q_{i,j}}+\left(1-p_{i,j}\right)log\frac{1-p_{i,j}}{1-q_{i,j}}$$

where the *p*_*i*,*j*_ denotes the memberships in the local fuzzy simplicial set, which was calculated based on the smooth nearest‐neighbor distances in the high‐dimensional space, the *q*_*i*,*j*_ represents the similarities between data points *i* and *j* in the reduced low‐dimensional space. Detailed explanation of *p*_*i*,*j*_ and *q*_*i*,*j*_ are referred to the ref. [44] The multi‐task learning loss function of FC module was designed according to four principles: a) Spots with similar metabolite profiles should be assigned to same region; b) Spots with spatially continuous should be assigned to same region; c) The number of spot categories should be as many as possible, to cape with the trivial solution; d) Spots from the same category in scribble‐inputting should be assigned to same region. Then, the loss function is designed as follows:

(8) $$\;L_{FC}=L_{sim}\;\left(r_{x,y},{\hat{o}}_{x,y}\right)+L_{tv}\left(r_{x,y}\right)+L_{ent}\left(r_{x,y}\right)+L_{scr}\left(r_{x,y},{\hat{s}}_{x,y}\right)$$

where the first term represents the error between the prediction and true value, as such $L_{sim}\;\left(r_{x,y},{\hat{o}}_{x,y}\right)=-\sum_{i=1}^{k}{\hat{o}}_{x,y,i}\log\left(r_{x,y,i}\right)$, where the $\hat{o}$ is the one‐hot encoding of the *O*, second term measures the spatial similarity between the input spot and its spatial neighbors, as such $L_{tv}\;\left(r_{x,y}\right)=∥r_{x+1,y}-r_{x,y}∥+∥r_{x,y+1}-r_{x,y}∥$; the third term serves as the penalty term to cope with the extreme case that the segmentation result only contains one single region, as such $L_{ent}\;\left(r_{x,y}\right)=-\frac{1}{k}\sum_{i=1}^{k}r_{x,y,i}\log r_{x,y,i}$; the fourth term measure the error between the prediction and the scribble category, as such $L_{scr}\;\left(r_{x,y},{\hat{s}}_{x,y}\right)=-\sum_{i=1}^{k}{\hat{s}}_{x,y,i}\log\left(r_{x,y,i}\right)$, where the $\hat{s}$ is the one‐hot encoding of the inputting scribble label *s*, which is only applied in scribble‐interactive mode. Curriculum learning was applied for training the GraphMSI model, where the DR module was first trained using *L_UMAP_* independently to warm up the model to generate the effective low‐dimensional embedding data, and then the FC module can be trained with fewer parameters as a slimmable neural network using the multi‐task loss function. The SGD optimizer with the 0.01 learning rate and the 0.9 momentum was set. In particular, the GraphMSI model with scribble‐interactive mode utilizes a pretrained model using unsupervised learning manner as its base. This base model was interactively fine‐tuned using scribble‐inputting to achieve enhanced segmentation results. Similarly, the GraphMSI model with the knowledge‐transfer mode uses model pre‐trained with the reference data to perform segmentation on unseen MSI data without re‐training the models. The model was developed in Python using the PyTorch library and trained on the workstation equipped with an Nvidia GTX 2080Ti GPU.

### Potential Molecular Marker Screening

The potential molecular markers identified from sub‐regions can assist in interpreting and validating the segmentation results of GraphMSI model. AUC was used as a quantitative metric to evaluate the classifier's effectiveness in distinguishing between classes, effectively summarizing the performance depicted by the receiver operating characteristic curve. A higher AUC value indicates better model performance in accurately differentiating between the designated positive class (the specified sub‐region) and the negative classes (other regions). For this purpose, logistic regression was employed as the classifier model.

### Performance Evaluation

Visual inspection and quantitative evaluation were both used to evaluate the performance of different segmentation methods. For visual inspection, the segmented sub‐regions were compared with H&E‐stained image and existing biological knowledge from the literature. For the quantitative evaluation, a Logistic regression model was constructed that uses lipids expressed in manually verified sub‐regions to predict their corresponding regions. Here, AUC was used to measure the correlation between the lipids and their associated sub‐regions, with a higher AUC value indicating more accurate segmentation results.

### Statistical Analysis

Data preprocessing and deep learning model construction were finished with Python 3.12.2 (e.g., transformation, normalization, evaluation of outliers). All results are reported as means ± SD based on five independent experiments. Statistical significance (*p < 0.05) was demonstrated by ANOVA test conducted in Python 3.12.2.

## Conflict of Interest

The authors declare no conflict of interest.

## Supporting information



Supporting Information

## Data Availability

The source code of the GraphMSI model together with the dataset for testing are available on https://github.com/gankLei‐X/GraphMSI.
