# Supplementary material for: Unraveling Spatial Heterogeneity in Mass Spectrometry Imaging Data with GraphMSI
Source: Adv Sci (Weinh). 2025 Jan 7;12(8):2410840. doi: 10.1002/advs.202410840 (PMC11848592; doi:10.1002/advs.202410840)
Supplement: Supplementary file 1 — Supporting Information [file ADVS-12-2410840-s001.docx]

Supporting Information

Unraveling Spatial Heterogeneity in Mass Spectrometry Imaging Data with GraphMSI

Lei Guo, Peisi Xie, Xionghui Shen, Ka-Yam Lam, Lingli Deng, Chengyi Xie, Xiangnan Xu, Kong Chu Wong, Jingjing Xu, Jiacheng Fang, Xiaoxiao Wang, Zhuang Xiong, Shangyi Luo, Jianing Wang, Jiyang Dong^*^, Zongwei Cai^*^

**E-mail:** [**zwcai@hkbu.edu.hk**](mailto:zwcai@hkbu.edu.hk)**, jydong@xmu.edu.cn**

**Abstract:** Mass spectrometry imaging (MSI) provides valuable insights into metabolic heterogeneity by capturing in situ molecular profiles within organisms. One challenge of MSI heterogeneity analysis is performing an objective segmentation to differentiate the biological tissue into distinct regions with unique characteristics. However, current methods struggle due to the insufficient incorporation of biological context and high computational demand. To address these challenges, we propose a novel deep learning-based approach, GraphMSI, which integrates metabolic profiles with spatial information to enhance MSI data analysis. Our comparative results demonstrate GraphMSI outperforms commonly used segmentation methods in both visual inspection and quantitative evaluation. Moreover, GraphMSI can incorporate partial or coarse biological contexts to improve segmentation results and enable more effective 3D MSI segmentation with reduced computational requirements. These are facilitated by two optional enhanced modes: scribble-interactive and knowledge-transfer. Numerous results demonstrate the robustness of these two modes, ensuring that GraphMSI consistently retains its capability to identify biologically relevant sub-regions in complex practical applications. We anticipate that GraphMSI will become a powerful tool for spatial heterogeneity analysis in MSI data.

DOI:


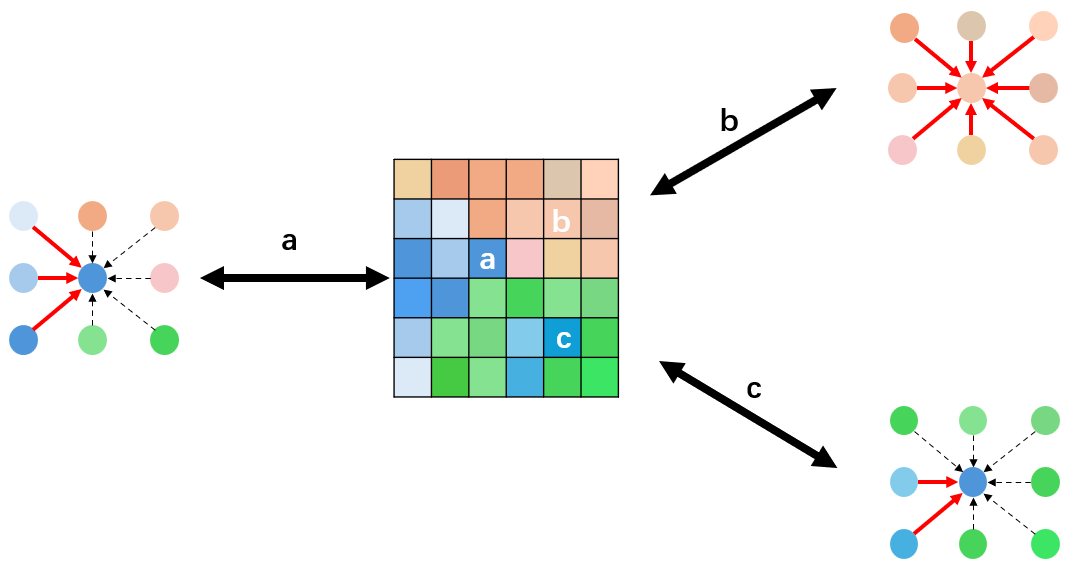


**Figure S1.** Detailed of GCN computation process in GraphMSI model, where spot with similar color exhibit comparable features. For efficient implementation, we used the "sparse" function in the "scipy" and "torch" packages to manage adjacency matrix storage and computations effectively. By leveraging sparse storage techniques, we were able to improve the computational efficiency of GraphMSI without compromising model performance.

**Figure S2.** Errors backpropagation diagram of GraphMSI.


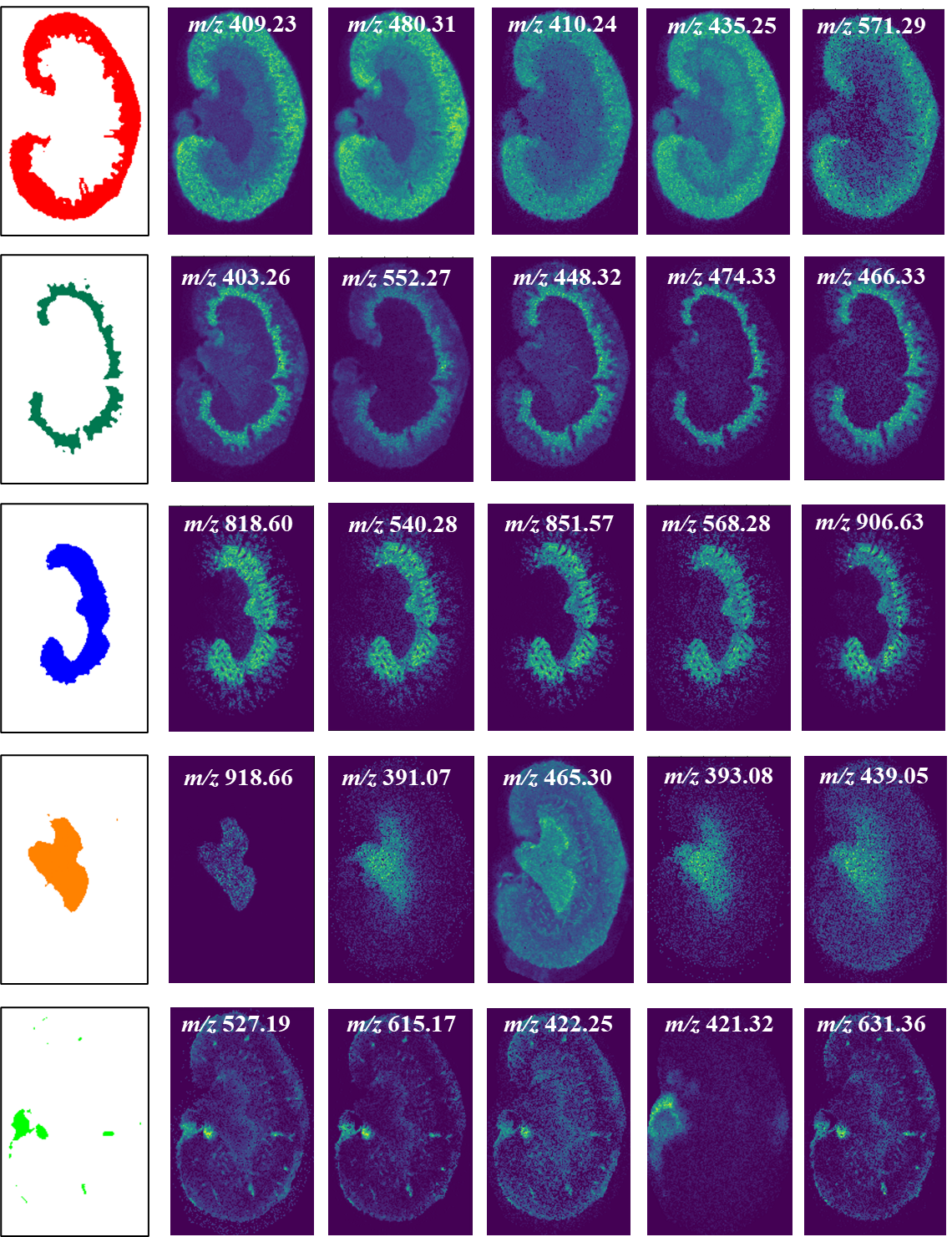


**Figure S3.** The searched five representative co-localized ions in outer cortex, inner cortex, renal medulla, renal pelvis, and an additional perirenal fat region.

**Figure S4.** Enhanced segmentation results on abdominal region in mouse fetus using GraphMSI with scribble-interactive mode. a) H&E-stained image; b) Color-encoded segmentation map derived from the basic of GraphMSI; c) Scatter plot of the entire data points in the UMAP embedding space related to (b); d) The created scribble and the corresponding segmentation results using scribble-interactive mode; e) Scatter plot of the abdominal region data points related to (d); f) Boxplot of Top 10 discriminative ions whose AUC > 0.70; g) Spatial distribution of discriminative ions.

**Figure S5.** Enhanced segmentation results on neck region in mouse fetus using GraphMSI with scribble-interactive mode. (A) H&E-stained image; (B) Color-encoded segmentation map derived from the basic of GraphMSI; (C) Scatter plot of the entire data points in the UMAP embedding space related to (B); (D) The created scribble and the corresponding segmentation results using scribble-interactive mode; (E) Scatter plot of the neck region data points related to (D); (F) Boxplot of Top 10 discriminative ions whose AUC > 0.70; (G) Spatial distribution of discriminative ions.


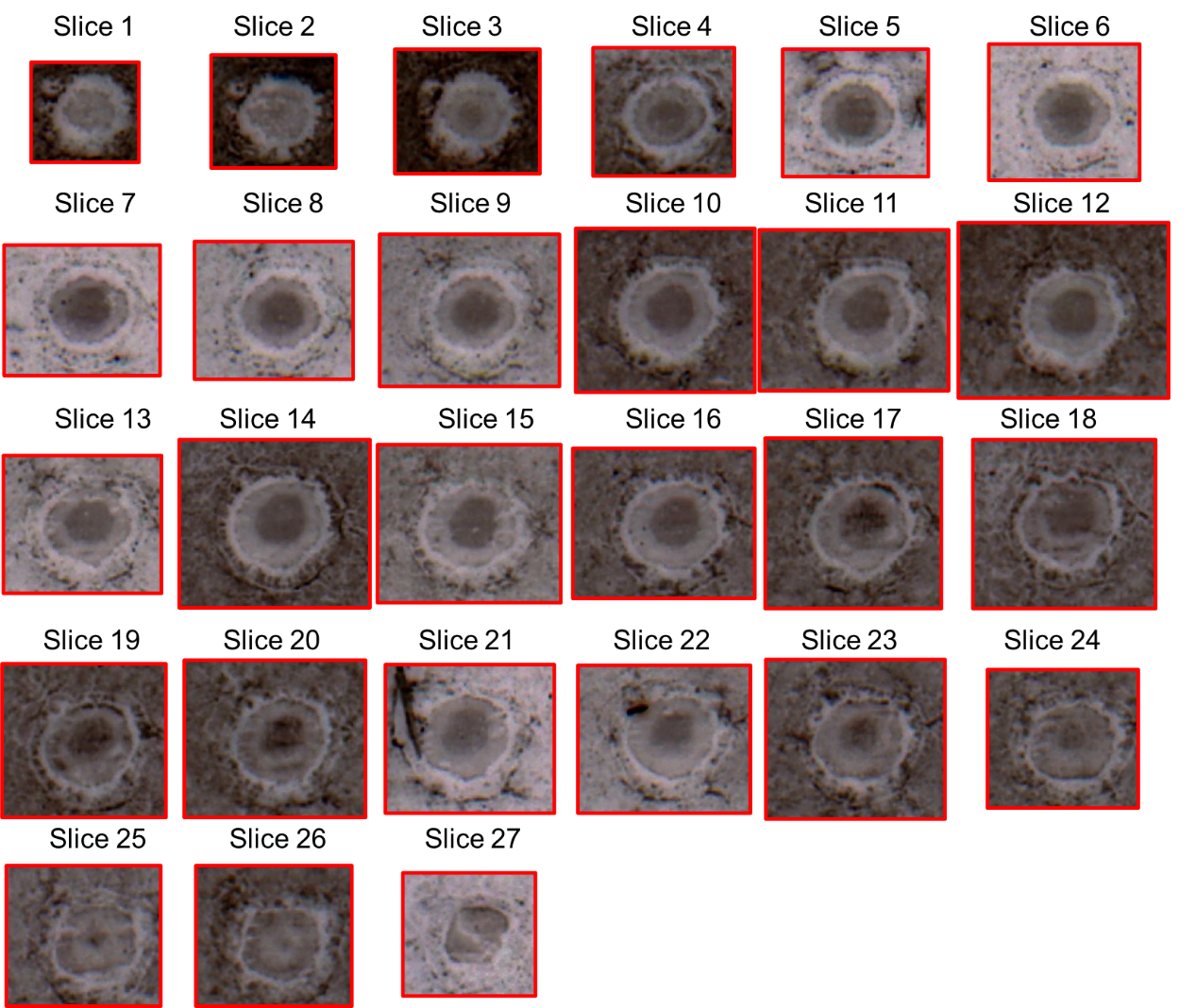


**Figure S6.** The optical images for the whole 27 CCS slices.


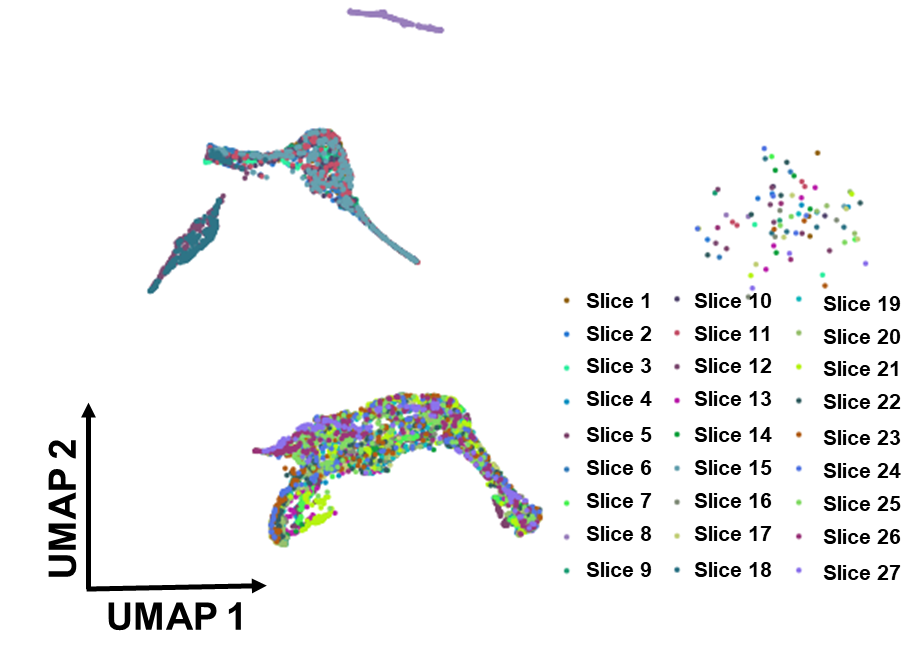


**Figure S7.** Scatter plot of data points in the UMAP embedding space colored by slice index.

**Figure S8.** Segmentation of the whole 27 slices using GraphMSI with knowledge transfer mode.

**Figure S9.** Scatter plot of data points in the UMAP embedding space, colored according to Figure S7.


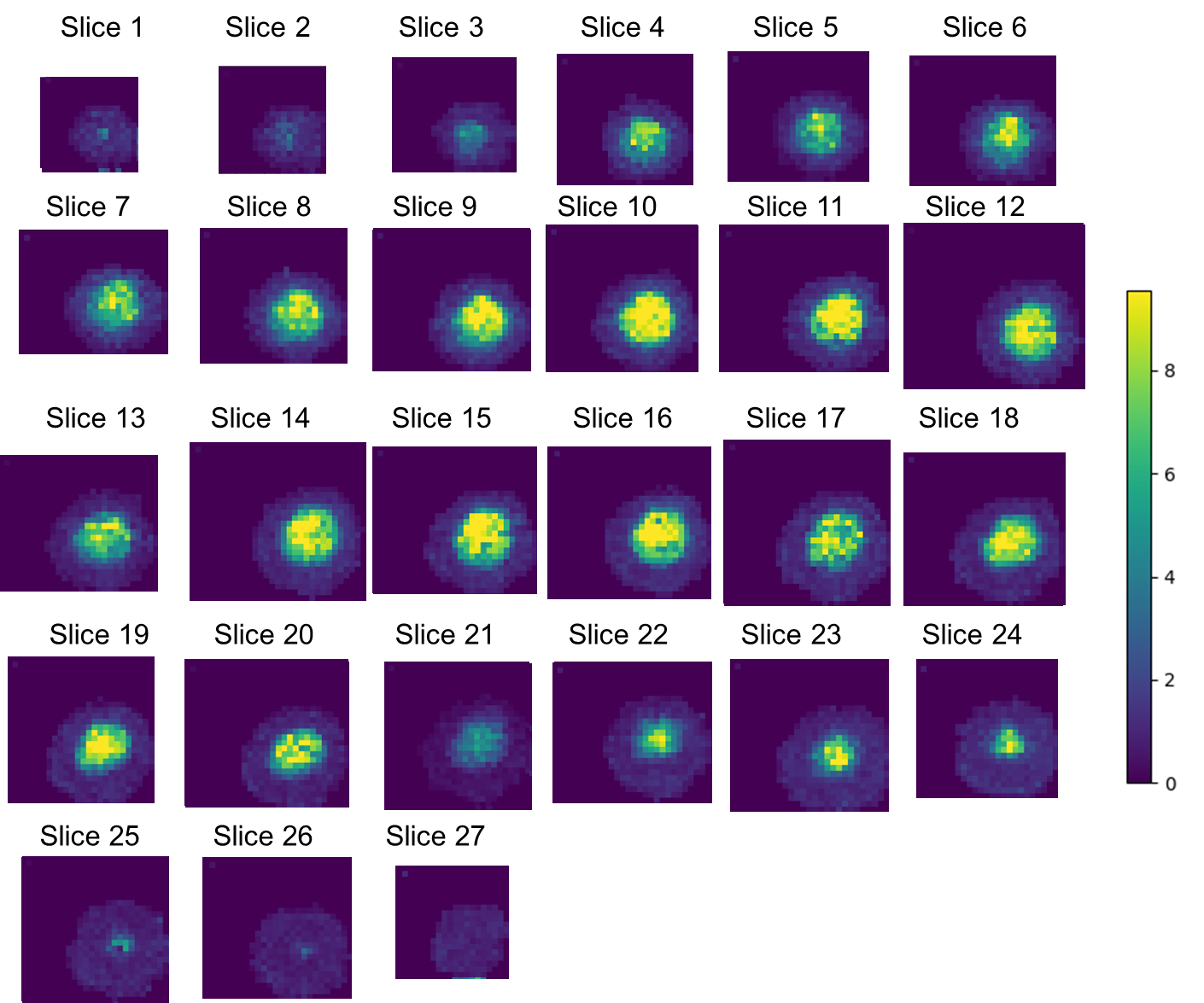


**Figure S10.** The spatial distribution of *m/z* 659.50 SM(d31:1) for the 27 CCS slices.


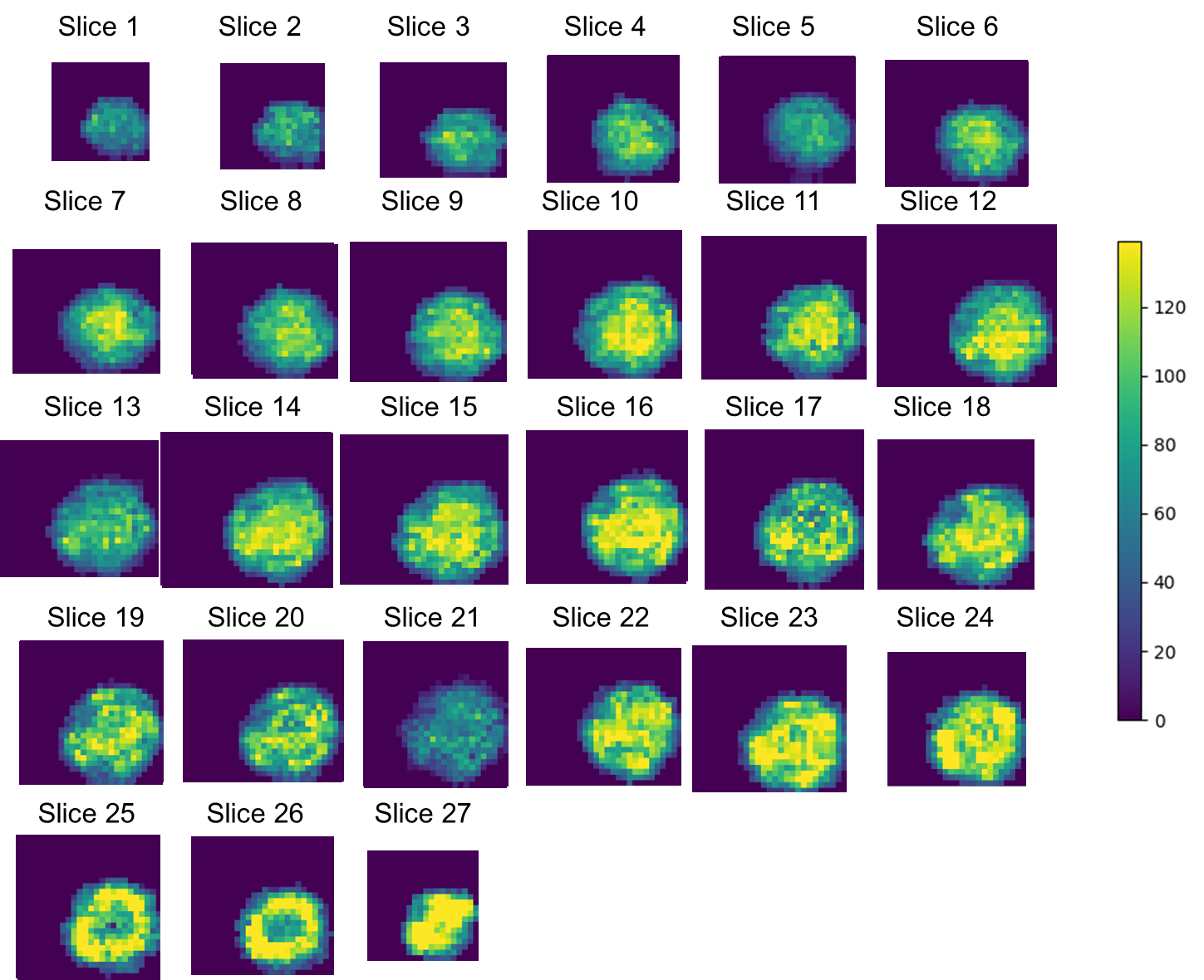


**Figure S11.** The spatial distribution of *m/z* 835.50 PI(34:1) for the 27 CCS slices.


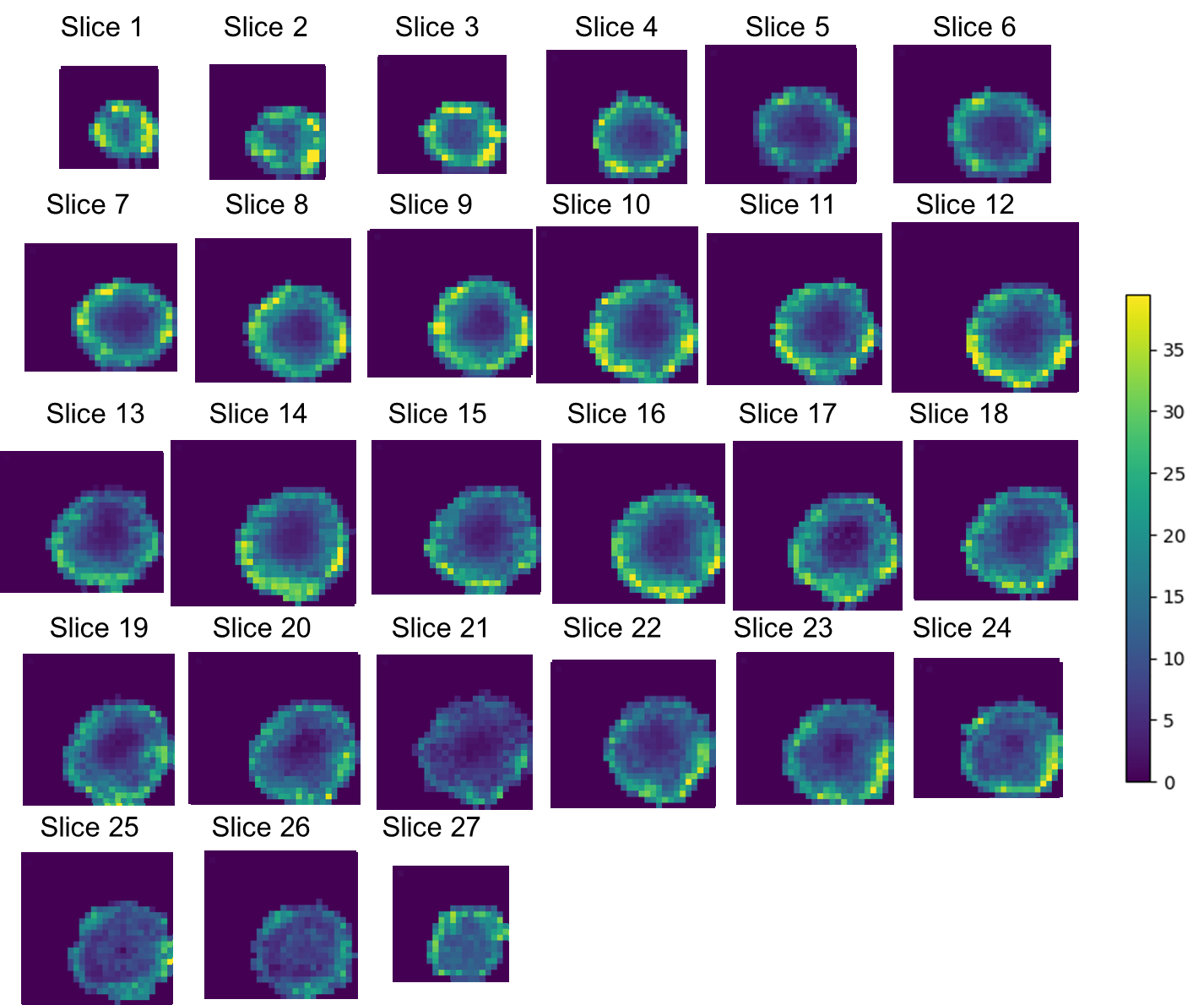


**Figure S12.** The spatial distribution of *m/z* 766.50 PE(38:4) for the 27 CCS slices.

**Figure S13.** Segmentation results obtained from t-SNE + K-Means, Cardinal pipeline, CNNAE+K-Means, CNN-based segmentation method and GraphMSI. a) Visual inspection results. b) Quantitative evaluation results.


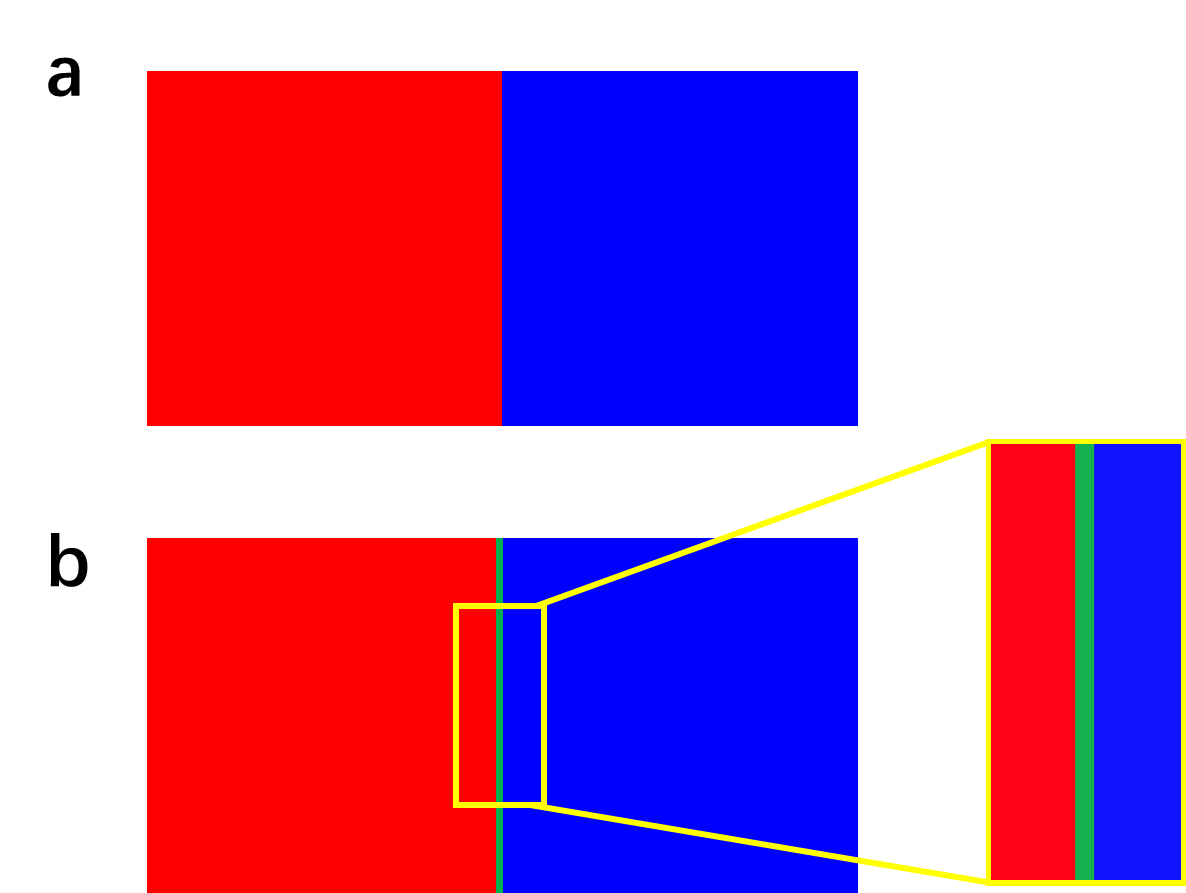


**Figure S14.** An illustration of the improper application of spatial information in MSI segmentation. (a) A simple case where the image contains only two regions. (b) The reconstruction data produced by methods that incorrectly utilize spatial information contains three values: red, green, and blue. Common feature extractors, such as CNNs, employ the sliding window technique to perform convolution operations on the original data. This process extracts both spectral and spatial features, aiming to reconstruct MSI data with enhanced spatial continuity. While this technique is effective in homogeneous regions, resulting in smooth segmentation, it tends to create artifacts at the boundaries. The reason is that while flat regions preserve feature consistency after convolution, the border areas—where two distinct flat regions intersect—undergo convolutions that deviate from those flat regions. This discrepancy leads to the generation of edge artifacts when using these commonly applied methods.


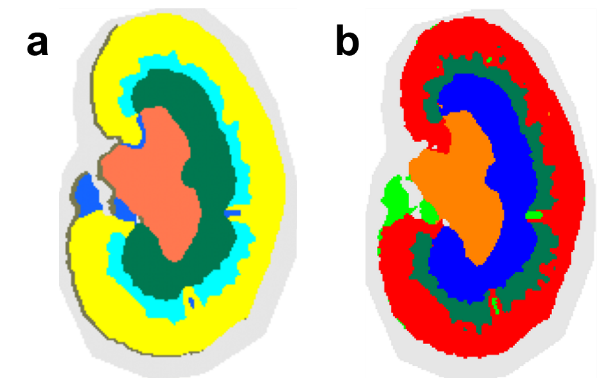


**Figure S15.** Segmentation results obtained from (a) CNN-based architecture and (b) GCN-based architecture of GraphMSI model.


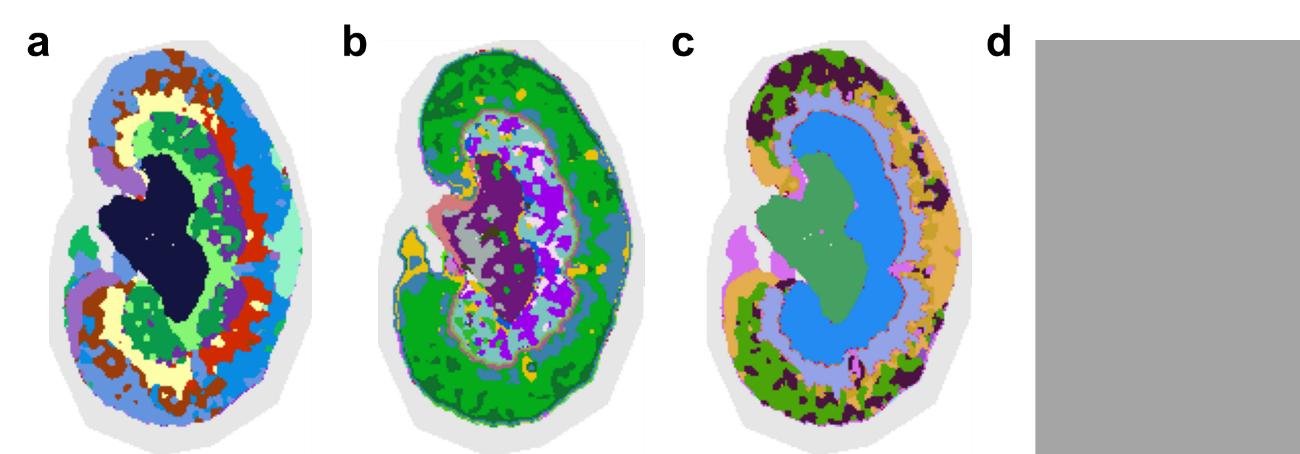


**Figure S16.** Segmentation results without (a) $L_{umap}$, (b) $L_{sim}$, (c) $L_{tv}$ and (d) $L_{ent}$ term in multi-task learning loss function.


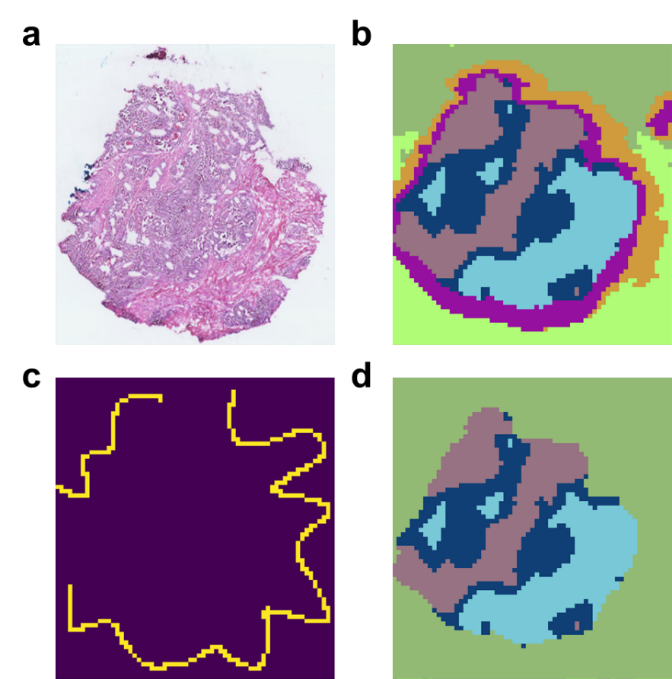


**Figure S17.** GraphMSI results on the human colorectal cancer dataset with 100µm spatial resolution. (a) H&E-stained image; (b) Color-encoded segmentation derived from GraphMSI with unsupervised learning; (c) Scribble-guided segmentation; (d) Segmentation results using scribble-interactive mode.


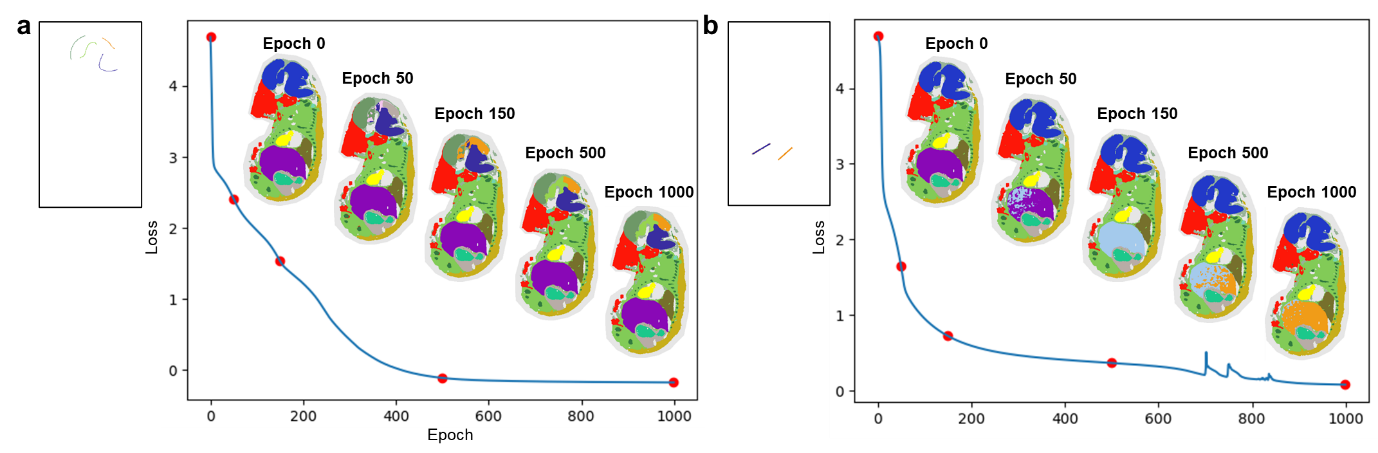


**Figure S18.** Loss value variation with epochs for (a) well-localized and (b) not well-localized scribbles in scribble-interactive mode.


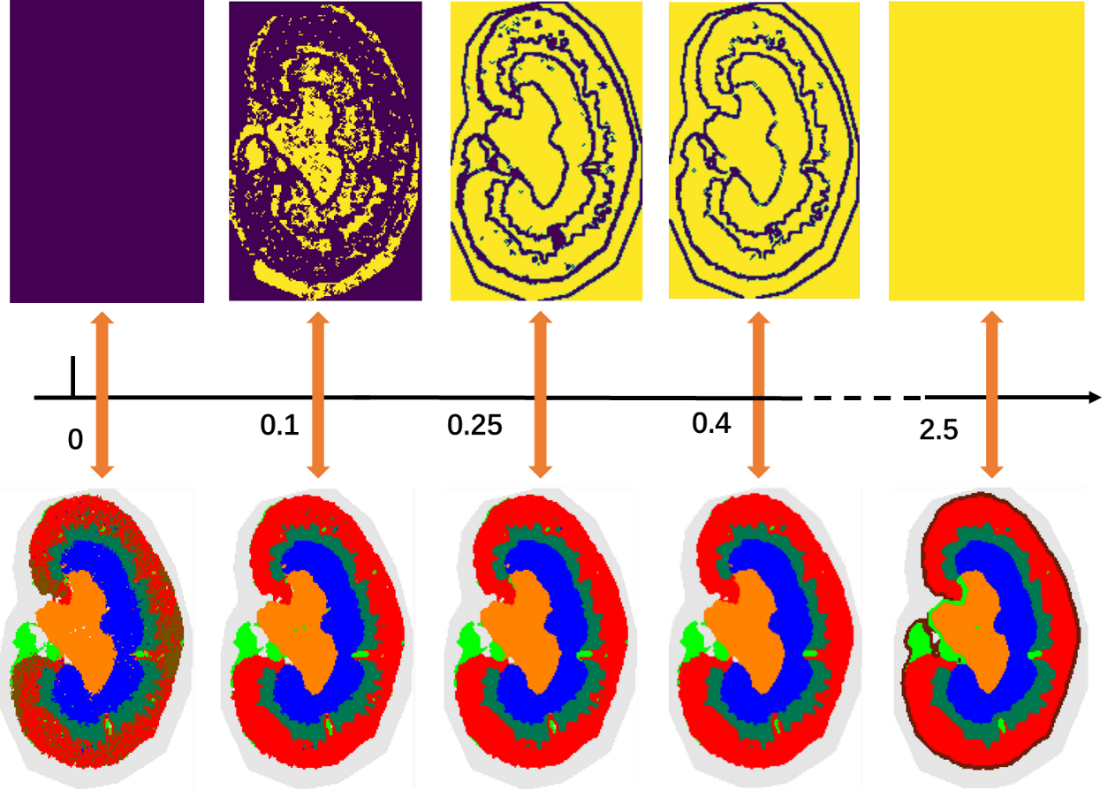


**Figure S19.** Segmentation results with different cut-off values.

**Table S1** The annotated discriminative ions from the Figure 3

| *m/z* | Calibration *m/z* | Metabolite | Adduct |
| --- | --- | --- | --- |
| 145.24 | 146.0447 | Glutamic acid | [M – H]- |
| 525.40 | 524.3138 | LPS(18:0) | [M – H]- |
| 766.47 | 766.5255 | PE(38:4) | [M – H]- |
| 810.53 | 810.5357 | PS (38:4) | [M – H]- |

**Table S2** The ANOVA test for different slices

| Feature | Slice 10 vs 11  p-value | Slice 10 vs 12  p-value | Slice 11 vs 12  p-value |
| --- | --- | --- | --- |
| UMAP 1 | 0.8518 | 8.462e-70 | 1.576e-71 |
| UMAP 2 | 0.8518 | 8.234e-85 | 5.060e-86 |

**Table S3.** Details about the training set and testing set of 3D CCS data

| **Training** | **Testing** |
| --- | --- |
| Slice 3 | Slice 1, 2 |
| Slice 5 | Slice 4, 6 |
| Slice 8 | Slice 7, 9 |
| Slice 11 | Slice 10, 12 |
| Slice 14 | Slice 13, 15 |
| Slice 17 | Slice 16, 18 |
| Slice 20 | Slice 19, 21 |
| Slice 23 | Slice 22, 24 |
| Slice 26 | Slice 25, 27 |

**Table S4.** Comparison of training parameters and running time between CNN-based model and GraphMSI model. Both models were configured with 3 layers and 100 kernels per layer, using a kernel size of 3×3 for the CNN.

| Tissue | Data Size | Model | Parameter Count | Running Time |
| --- | --- | --- | --- | --- |
| Mouse Kidney | 132 $\times$ 205 | CNN-based model | 28600 | 6.01s $\pm$ 0.14s |
|  |  | GraphMSI | 22200 | 12.7s $\pm$ 0.32s |
| Mouse Fetus | 437 $\times$ 241 | CNN-based model | 28600 | 7.50s $\pm$ 0.31s |
|  |  | GraphMSI | 22200 | 21.51s $\pm$ 0.74s |

**Section S1. The roubust of GraphMSI with scribble-interactive mode**

We have presented the results of GraphMSI with scribble-interactive mode for high spatial resolution datasets, a 50µm fetus mouse datase, where GraphMSI demonstrated high segmentation accuracy. To further explore its performance at lower spatial resolutions, we conducted an additional experiment on a public human colorectal cancer dataset with a 100µm spatial resolution^[1]^. As shown in the **Figure S17**, we used the scribble-interactive mode in GraphMSI, guided by scribbles created based on the H&E-stained image (**Figure S17a**). Even at this lower spatial resolution, the model effectively aggregated meaningless matrix regions and preserved sub-regions related to tumor areas (**Figure S17d**), These results suggest that GraphMSI performs robustly at both high and moderate spatial resolutions, maintaining its ability to distinguish biologically relevant sub-regions.

To assess the robustness of the scribble annotations, we conducted an experiment on the mouse fetus dataset, where we used two types of scribble inputs: one with well-localized scribbles aligned with the optical images and another with scribbles that were not precisely localized. We observed that with well-localized scribbles, the model’s loss function optimized smoothly with increasing epochs, and the resulting segmentation maintained consistent and rational boundaries across all sub-regions (**Figure S18a**). Conversely, when using less accurately localized scribbles, the model's loss fluctuated erratically during training and had minimal impact on the final segmentation (**Figure S18b**). This robustness arises from the feature similarity and scribble regularization incorporated in GraphMSI, which allow the model to adjust segmentations based on the internal consistency of biological tissue, even if the input scribble information does not perfectly align. These results demonstrate the robustness of GraphMSI with the scribble-interactive mode.

**Section S2.** **The impact of cut-off value on the segmentation results**

The graph construction strategy plays the critical role in GraphMSI segmentation, especially concerning the user-defined cut-off value for edge weight. **Figure S19** demonstrates the segmentation results achieved with varying cut-off values. A cut-off value that is too low (threshold = 0) introduces minimal spatial information, leading to discontinuous segmentation. Conversely, a cut-off value that is too high (threshold = 2.5) results in continuous segmentation but with significant edge artifacts. Adaptive cut-off values (thresholds = 0.1, 0.25, 0.4) not only ensure continuous segmentation but also prevent the occurrence of edge artifacts. To facilitate this, we have developed an interactive graphical user interface (GUI) for GraphMSI that aids in determining the appropriate cut-off for effective segmentation. Specifically, we create a blank picture, and nodes with a degree less than 4 are considered potential edge nodes. Subsequently, the user can slide the bar to select the appropriate cut-off value to obtain visualized results of the edge nodes. Based on our experience, when the visualized results of the edge nodes roughly match the structures from H&E, optimal segmentation results can be achieved. It is worth noting that we have a significant tolerance range for the optimal cut-off value. In the MSI data of mouse kidney, cut-off values between 0.1 and 0.4 provide good segmentation results.

**Section S3 Experiment details for mouse kidney and mouse fetus**

**Animal experiments.** The mice are kept in a sterile environment with individually ventilated cages, following a 12-hour light/dark cycle at a temperature of 22°C and a relative humidity of 45%. Pregnant mice are housed separately. They have unrestricted access to food and water under standard conditions. Kidney tissues and entire GD 14.5 fetus tissues are collected from 23-week-old mice and naturally pregnant mice, respectively. All samples are promptly stored at -80°C for future experiments.

**Histological staining.** The tissues are sectioned at a thickness of 10 μm using a CryoStar NX70 cryostat (Thermo Fisher, Waltham) and then stained with hematoxylin and eosin (H&E). The staining protocol involves the following steps: a quick rinse in water (2 seconds); immersion in hematoxylin (3 minutes); rinsing under running water (1 minute); a brief dip in ethanol with 1% HCl (2 seconds); another rinse in running water (3 minutes); immersion in eosin (6 seconds); and a final rinse in running water (3 minutes)

**Sample preparation.** Tissue samples are sectioned to a thickness of 10 µm and placed on ITO coated slides (Delta Technologies, USA). Following mounting, the slides are dried in a vacuum desiccator for 30 minutes. Matrix application is performed using an HTX M5 Sprayer™ (HTX Technologies, USA). The matrix solution is prepared with 7 mg/mL NEDC in 70% methanol. The spraying process is conducted at 10 psi nitrogen pressure, with a flow rate of 80 µL/min over 6 cycles, at a spray temperature of 65°C. After spraying, the samples are further dried in a vacuum desiccator for at least 60 minutes to be ready for MALDI-MSI analysis.

**Data acquisition.** For the mouse kidney, data is acquired using the timsTOF fleX MALDI-2 in negative ion mode. The mass range is set to *m/z* 50-1050 with a resolution of 50 µm using the M5 small mode. The laser operates at a frequency of 10,000 Hz with a power of -90%, delivering 300 laser shots. Other optimized instrument settings include funnel 1 RF at 500 Vpp, transfer time of 110 µs, multipole RF at 1000 Vpp, pre-pulse storage of 10 µs, funnel 2 RF at 500 Vpp, and collision RF at 1200 Vpp, are utilized to generate MSI data. For the mouse fetus, data is collected using the RapifleX MALDI-TOF in negative ion mode, with a mass range of *m/z* 20-1000 and a resolution of 30 µm in single laser mode, with 60 laser shots. The remaining instrument parameters are set to their default values. Both of two datasets are acquired using flexControl (Bruker, Germany) and flexImaging (Bruker, Germany).

**Section S4 Experiment details for** **cancer cell spheroids**

**Sample preparation.** HCT116 colorectal carcinoma cells (ATCC, Manassas, VA, USA) are cultured in GRM1640 complete medium (Thermo Fisher Scientific, USA). Cell culture, spheroids formation and sample sectioning follow the protocol outlined in previous works. ^[2, 3]^

**Data acquisition.** MSI data acquisition is performed using the RapifleX MALDI-TOF in negative ion mode. Mass spectra are obtained using 1000 laser shots and a lateral resolution of 50 μm in M5 small mode, covering a mass range of *m/z* 100–1000. The laser is operated at 88% power, with a detector gain of 3 × 3352 V and a repetition rate of 10000 Hz. The remaining instrument parameters are set to their default values.

**References**

[1] P. Inglese, J. S. McKenzie, M. Anna, J. Kinross, K. Veselkov, E. Holmes, Z. Takats, J. K. Nicholson, R. C. Glen, Chem. Sci. 2017, 8, 3500-3511.

[2] P. Xie, X. Liang, Y. Song, Z. Cai, Anal. Chem. 2020, 92, 11341.

[3] G. J. LaBonia, K. R. Ludwig, C. B. Mousseau, A. B. Hummon, Anal. Chem. 2018, 90, 1423.
